# A New Scoring Model to Diagnose COVID-19 Using Lung Ultrasound in the Emergency Department

**DOI:** 10.1186/s43168-021-00102-w

**Published:** 2022-02-01

**Authors:** Mohammad Eltahlawi, Hesham Roshdy, Mohammad Walaa, Panagiota Manthou, Diego Araiza Garaygordobil, Mohammad Elshabrawy, Mohamed Elkholy, Mohammad Abdelkhalek Basha, Marwa Tharwat, Waleed Mansour

**Affiliations:** 1 Cardiology, Faculty of Medicine, Zagazig University, Zagazig, Egypt; 2 Chest, Faculty of Medicine, Zagazig University, Zagazig, Egypt; 3 Deputy Coordinator of Emergency and Intensive Care Nursing Speciality, National Kapodistrian University of Athens, Athens, Greece; 4 National Institute of Cardiology, México, México; 5 Department of Agricultural Economics & Statistics, Faculty of Agriculture, Zagazig University, Zagazig, Egypt; 6 Radiology, Faculty of Medicine, Zagazig University, Zagazig, Egypt; 7 Anatomy, Faculty of Medicine, Zagazig University, Zagazig, Egypt; 8 Chest Diseases, Faculty of Medicine Zagazig University, Zagazig, Egypt

**Keywords:** COVID-19, SARS-CoV-2, Lung ultrasound, Corona Virus, Pneumonia, New Score

## Abstract

**Background:**

Several studies have reported the predictors of the prognosis in COVID-19 patients; however, smoking, X-ray findings of pulmonary congestion, and A-profile and areas of consolidation in LUS are independent predictors for COVID-19 infection. The new score had a sensitivity of 93.8% and a specificity of 58% for the prediction of COVID-19. Mortality in COVID-19 patients is significantly correlated with age, fever duration, cardiac history, and B-profile and areas of consolidation in LUS. However, it is negatively correlated with initial O_2_ saturation and ejection fraction. This study aimed to design a new scoring model to diagnose COVID-19 using bedside lung ultrasound (LUS) in the emergency department (ED).

**Results:**

Eighty-two patients were recruited. Fifty patients (61%) were negative for COVID-19, and 32 (39%) were positive. Sixty-four patients (78%) recovered while 18 patients (22%) died. COVID-19 patients had more AB-profile and more areas of consolidation than the non-COVID-19 group (*p*<0.001). Smoking, congestion in X-ray, A-profile, and abnormal A line in LUS are independent predictors for COVID-19 infection. The score had a sensitivity of 93.8% and a specificity of 58% for the prediction of COVID-19. Mortality in COVID-19 patients is significantly correlated with age, fever duration, cardiac history, and B-profile and areas of consolidation in LUS. However, it is negatively correlated with initial O_2_ saturation and ejection fraction.

**Conclusions:**

In conclusion, the application of our new score can stratify patients presented to ED with suspected COVID-19 pneumonia, considering that it is a good negative test. Moreover, this score may have a good impact on the safety of medical personnel.

**Trial registration:**

ClinicalTrials.gov Identifier: NCT05077202. Registered October 14, 2021 - Retrospectively registered, https://clinicaltrials.gov/ct2/show/NCT05077202

## Background

Coronavirus disease 2019 (COVID-19) pandemic is the most serious medical problem worldwide nowadays. One of the primary findings for COVID-19 is pneumonia [1, 2]. Many cases with chest infection may have similar symptoms and signs to COVID-19 infection. Real-time reverse-transcriptase polymerase chain reaction (RT-PCR) assay is the test widely used to diagnose COVID-19 [3]. However, RT-PCR test results of pharyngeal swab specimens have some variability and potential instability; therefore it should not be considered as the only indicator for diagnosis [4]

Although COVID-19 infected patients have some typical radiological computed tomography (CT) findings even in asymptomatic patients [5], atypical findings are increasingly common. Several studies have reported the predictors of the prognosis in COVID-19 patients [6, 7]. Lung ultrasound (LUS) has been widely considered over the last years due to its medical and surgical value [8, 9]. The role of LUS in the care of patients with critical respiratory conditions is nowadays widely documented [10]. LUS can help in reducing the number of health care professionals exposed during patient stratification by simple, rapid bedside test [11, 12]. LUS could be done without patient immobilization that might spread the infection. This differs from CT chest that requires patient mobilization and may be difficult in critical ventilated patients, in addition to its radiation hazards and high cost.

Many patients present to the emergency department (ED) with severe chest symptoms that need to be investigated for COVID-19 before transfer to the intensive care unit (ICU). The timely transfer of patients to ICU or a designated unit (isolation or quarantine) with sufficient rescue equipment should be considered even if their of RT-PCR test results for pharyngeal swab specimens are negative to limit the spread of infection. Therefore, the need for rapid bedside diagnostic tools is highly appreciated. In this study, we aimed to find a simple bedside investigations that could suspect the diagnosis and/or predict the prognosis of COVID-19 in ED before in-hospital admission of the patient.

## Methods

### Study design

The study is an international multicentre observational study that included three centres (BLINDED).

### Ethical statement

After approval from the institutional review boards (IRB) of the faculty of medicine, Zagazig University ( No., 6930), all patients were properly counselled and signed informed written consent.

### Patients

The study recruited all patients with pulmonary symptoms and attended to ED between 27^th^ March 2020 and 17^th^ May 2020. Exclusion criteria were (i) patients with congestive heart failure (*n*= 7), (ii) patients with known interstitial lung fibrosis and any chronic pulmonary disease (*n*= 4), and (iii) patients with poor echo-window (*n*= 4). Patients were seen first at ED, where they underwent the required investigations and then classified. Patients were questioned about symptoms suspecting COVID-19 infection. Those who met the suspected clinical and investigational criteria were given a standard mask and were rapidly transferred safely to a separate waiting and isolation area with available infrastructure and tools for hand and respiratory hygiene practice. If the patient was proved to be positive for COVID-19 according to RT-PCR assay, he was sent to quarantine. For negative patients, they were admitted to intermediate or ICU according to their clinical status. All recruited patients underwent the following: complete blood count (CBC), arterial blood gas (ABGs), RT-PCR assay to detect COVID-19, chest X-ray, chest CT, LUS, and echocardiography (according to its availability, with precautions for the operators and the probe similar to those exerted to LUS).

### LUS examination

Two trained medical personnel, one ICU physician and one ICU nurse, entered the isolation room respecting all the preventive measures for respiratory, droplet, and contact isolation provided by the world health organization for the COVID-19 outbreak. The ultrasound probe and the tablet were put in two different sterile plastic probes and tablet covers. Imaging was performed using a curvilinear probe (2–5 MHz) with different devices according to the availability in each centre. Six-point LUS (three in each hemithorax) was performed as described in the bedside lung ultrasonography in emergency (BLUE) protocol [13].

### Statistical analysis

Statistical analysis included comparing different parameters between COVID-19 positive-patients and COVID-19 negative-patients, using independent t-test for numerical variables and chi-square for categorical variables. All significantly different variables were entered in a forward stepwise binary logistic regression analysis to select the best model. After selecting the best model. The variable chosen in the last step was weighed using the odds ratios (ORs) calculated from the regression coefficient (β) for each variable, the ORs were multiplied by 0.125 to calculate a score for each variable and the number was rounded to the nearest integer giving of scoring system of 10 points. All study patients were scored. The cutoff point of the score was calculated using ROC analysis, and calculation of sensitivity and specificity was performed. Also, variables associated with mortality in COVID-19 positive were entered in a forward binary logistic regression, which selected the best model, and the ORs were calculated for each variable using the regression coefficient (β). Before adding the variables in the regression analysis, the proper cutoff values of different contenious variables were determined using ROC analysis. Patient Data were entered, checked, and analyzed using SPSS for Windows version 16 (SPSS, Inc. Chicago, IL, USA). For all the above mentioned statistical tests, the threshold of significance is fixed at a 5% level (*p* < 0.05).

## Results

### Patients

Eighty-two patients were recruited (50 males, 32 females; mean age, 57.2 ± 15.23 years). Fifty patients (61%) were negative for COVID-19, while 32 (39%) were positive. The mean hospital stay was 14 ± 4.14 days. Sixty-four patients (78%) were recovered and discharged to home, and 18 patients (22%) died within 2 weeks of follow-up. There was a significant difference between COVID-19 and non- COVID-19 patients regarding smoking (*p* = 0.01), while there was no significant difference between both groups regarding sex, diabetes, hypertension, and cardiac disease (Table 1).

**Table 1:** The patients’ demographic and clinical data

Variable	COVID-19		*P*-Value
	Positive (*n*= 32)	Negative (*n*= 50)	
Age, years, mean ± SD	58.8 ± 16.47	56.2 ± 14.45	0.45
Sex			0.082
Male	23 (71.9)	27 (54)	
Female	9 (28.1)	23 (46)	
Smoking	20 (62.5)	17 (34)	0.011
Diabetes	9 (28.1)	20 (40)	0.272
Cardiac/hypertensive	18 (56.2)	23 (46)	0.365
Fever	28 (87)	43 (86)	0.845
Duration of fever, days, mean ± SD	5.3 ± 1.58	3.7 ± 1.27	<0.001
Admission time, days, mean ± SD	15 ± 5.82	13.4 ± 2.43	<0.001
O2 saturation ABG, mean ± SD	0.8 ± 0.12	0.9 ± 0.05	<0.001
PH in ABG, mean ± SD	7.4 ± 0.05	7.4 ± 0.05	0.949
Lymphocytic count (X10^3^) , mean ± SD	14.6 ± 8.78	30.4 ± 13.62	<0.001
Mortality	12 (37)	6 (12)	0.006

Unless otherwise indicated, data represent the number of patents with percentage in parenthesis

*n* number, *SD* standard deviation

### Imaging findings

**Fig. 1:**
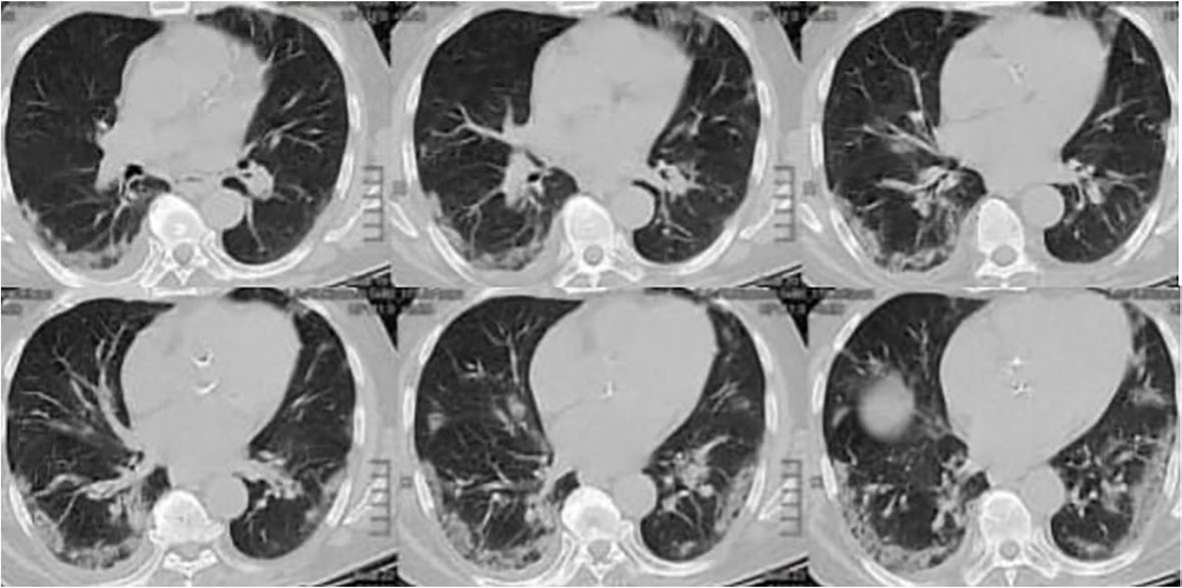
A 62-year-old man restaurant owner was admitted to the emergency room (ER) with a 3-day history of fever (38.2°C/100.8°F) and dyspnea. He reported no other symptoms, nor had a history of travels abroad nor exposure to patients infected or suspected of contagious COVID-19. Xray findings: consolidation with linear brochogram in both lobes us findings: very thick pleural line, Multiple B-lines EF: 40-45% CT findings: GGO and massive consolidation in the posterior parts of the lower lobes. Lymphocytes when he inserted in ICU: 6, 62% troponine at 1st 24h in ICU: 3.2 pg/n. He was confirmed to have Covid19 by PCR

**Fig. 2:**
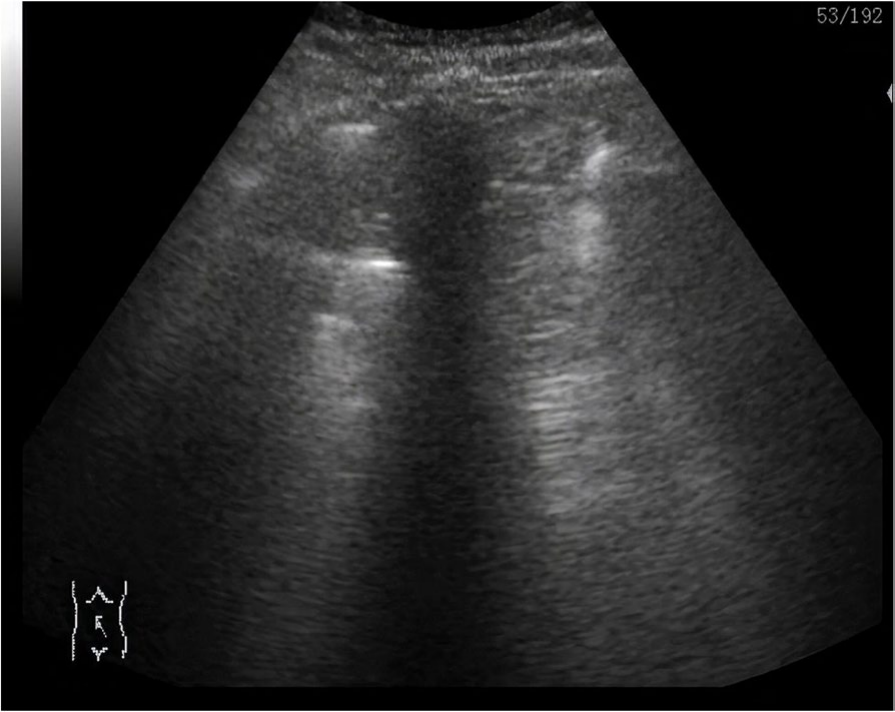
The second patient is 43-year-old male patient complaining of fever for 3 days associated with cough, symptoms of pharyngitis and dyspnea. He is a moderate smoker. He was telling a history of close contact with Covid-19 infected patient. He was examined in ER, and was hemodynamically stable but with high temperature (38.9° C) and with O2 saturation of 91%. Chest X ray revealed bilateral reticulo-nodular infiltrates in both middle and lower lung lobes. Lung ULS showed areas of consolidation and thick smooth pleural line. B-lines were few and dispersed and there was no pleural effusion. He has low score of suspicion and proved to be non-Covid after 3 PCR swabs

**Fig. 3:**
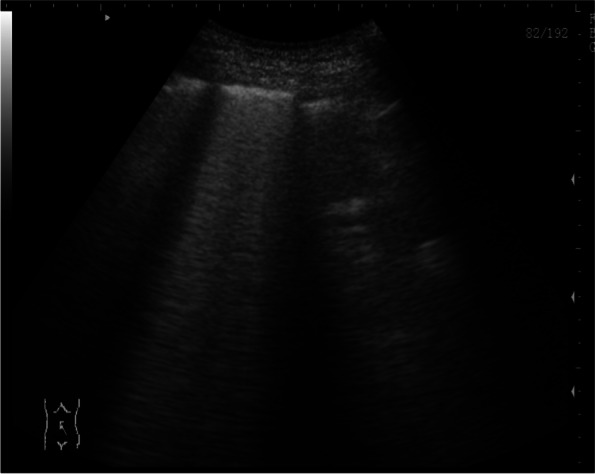
LUS of a non-COVID-19 patient showed areas of consolidation with small areas of air bronchogram and thick smooth pleural line. B-lines were few and not so confluent

There was a highly significant difference between both groups in the context of lung congestion in X-ray with more congestion profile in COVID-19 patients (*p* = 0.0004). In CT chest, there was more consolidation and ground-glass appearance among COVID-19 positive-patients (*p* < 0.001) (Fig. 1), while they had less pleural effusion than COVID-19 negative-patients (*p* = 0.026). Concerning LUS, there were more areas of consolidation in the COVID-19 positive group (*p* < 0.001). There was no significant difference between both groups regarding the number of B lines per SLF (*p* = 0.17), while there was a significant difference between both groups concerning the distance between B lines with closer, more confluent lines in COVID-19 related pneumonia (*p* < 0.001) (Table 2) (Figs. 2 and 3). There was a negative correlation between the distance of B lines on one side and ground glass/ consolidation by CT on the other side (*p* < 0.01)

**Table 2:** Imaging findings

Variable	COVID-19		*P*-Value
	Positive (*n*= 32)	Negative (*n*= 50)	
X-ray			
Normal	5 (15.6)	23 (46)	0.005
Congestion	30 (93)	29 (58)	0.0004
Consolidation	8 (25)	13 (26)	0.919
Plural effusion	0 (0)	2 (4)	0.252
Pneumothorax/Hydropneumothorax	1 (3.1)	6 (12)	0.160
B lines / SLF, mean ± SD	4.6 ± 1.19	4.2±1.33	0.172
Distance between B lines, mm, mean ± SD	3.6 ± 0.87	4.9 ± 1.14	<0.001
CT			
Normal	1 (3.1)	0 (0)	0.209
Consolidation	13 (40.6)	50 (100)	<0.001
Effusion	0 (0)	7 (14)	0.026
Ground glass opacity	15 (46.9)	0 (0)	<0.001
Echocardiography			
Ejection fraction^a^, mean ± SD	0.5 ± 0.09	0.5 ± 0.12	0.703
Valvular affection^a^	5 (20.8)	1 (11.1)	0.518
Pericardial affection^a^	4 (16.7)	2 (22.2)	0.712
Ultrasound			
A- lung ultrasound pattern	28 (87)	18 (36)	<0.001
B- lung ultrasound pattern	6 (18.8)	35 (70)	<0.001
C- lung ultrasound pattern	5 (15.6)	10 (20)	0.617
Abnormal A line	26 (81.2)	17 (34)	<0.001
Thick plural line	28 (87.5)	42 (84)	0.662
Interrupted plural line	14 (43)	17 (34)	0.374
Smooth plural line	1 (3.1)	9 (18)	0.044
Alveolar consolidation	21 (65)	25 (50)	0.164
Plural effusion	7 (21.9)	15 (30)	0.418

Unless otherwise indicated, data represent the number of patents with percentage in parenthesis

^a^: number of Echo done *n*=24 in COVID19 positive and *n*=10 in COVID-19 negative; *n* number, *SD* standard deviation

### Validity of new score

Linear regression analysis for the independent predictors for being COVID-19 positive was done (Table 3). Smoking, congestion in x-ray, LUS profile-A, and abnormal A line in LUS were detected to be significant independent predictors for COVID-19 infection. We considered abnormal A lines in (A thick pleural line that may suggest findings such as pneumonia, ARDS, or fibrosis. In addition, an irregular pleural line (Interrupted dense artifact) that is pathologic and may suggest pulmonary fibrosis or pneumonia.

**Table 3:** Variables in the regression model

Variable	B	SE	Wald	df	Sig.	Exp (B)	95% CI for EXP (B)
Smoking	2.04939	1.011	0.34	1	<0.001	4.2	0.161-8.495
Abnormal A line in LUS	2.90792	0.062	1.139	1	<0.001	8.45	1.23-15.23
Congestion in X-ray	3.628429	2.64E+03	0.23	1	<0.001	13.2	1.233-33.23
LUS profile-A	4.334467	24.59	0.64	1	<0.001	18.78	2.334-25.345
Constant	0.587707	1.68E+04	0.034	1	0.994	0.3454	

*LUS* lung ultrasound, *B* coefficient for the constant, *SE* standard error, *Wald* Wald chi-square test, *df* degree of freedom, *Sig*. *P*-value, *Exp (B)* exponentiation of the B coefficient, which is an odds ratio, *CI* confidence interval

**Fig. 4:**
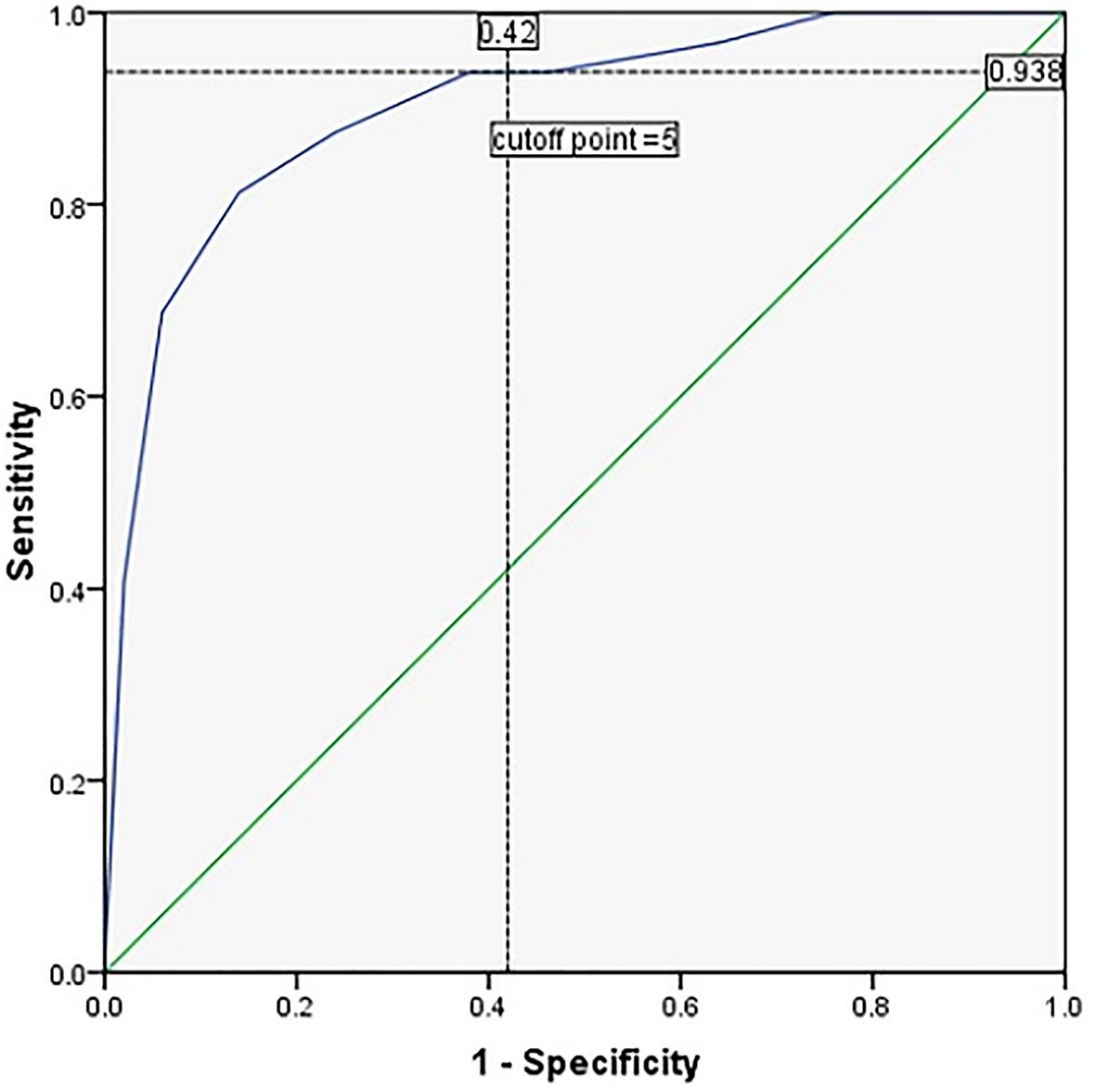
ROC curve constructed to determine the cutoff value of the score with a sensitivity of 93.8% and a specificity of 58% in detecting COVID-19 positive patients

A scoring system for the prediction of COVID-19 diagnosis using clinical and radiological data was created. According to the odds ratio from the regression equation, smoking was given 1 point, abnormal A line was given 2 points, congestion in x-ray was given 3 points, and LUS profile-A was given 4 points. We analyzed the data set of our score to determine the best score for predicting COVID-19 diagnosis using the ROC curve. ROC analyses (Fig. 4) yielded an ideal score of >5 for detecting COVID-19 positive-patients (AUC: 0.902, 95% CI: 0.883- 0.971, *p* <0.001). The application of this score was associated with a sensitivity of 93.8% and specificity of 58%.

### Correlation in COVID-19 positive-patients

By performing the correlation between clinical, laboratory, and radiological factors in COVID-19 positive-patients on one side and mortality on the other side (Table 4), we found that there was a significant positive correlation between mortality and each of age, duration of fever, presence of cardiac disease including hypertension, and presence of B-profile, and areas of consolidation in LUS. There was a significant negative correlation between mortality and initial O_2_ saturation and ejection fraction (EF) in echocardiography.

**Table 4:** Correlation in COVID-19 positive patients

Variable	r	*P*-value
Age	0.691^b^	>.0001
Days of fever	0.440^a^	.012
Cardiac including hypertension	0.423^a^	0.016
Ejection fraction	-0.694^b^	>.0001
O2 saturation	-0.598^b^	>.0001
Profile-B in LUS	-0.415^a^	0.018
Abnormal A line	0.372^a^	0.036

*LUS* lung ultrasound, *r* Pearson coefficient

^a^: significant

^b^: highly significant

## Discussion

Due to the lack of a standardized score for COVID-19 patients, some clinicians depend on other previously validated scores [14], such as the lung ultrasound aeration score [15]. We designed a new scoring model including four items and tested its validity in predicting COVID-19 using bedside LUS in the ED. We found that all four items of our score were significant independent predictors for COVID-19 infection by linear regression analysis. This score has high sensitivity (93.8%) in detecting COVID-19 infection, making it a good negative test. In patients with suspected COVID-19 or those presented to ED, applying this new score help physicians exclude those non-infected patients as a first screening step before dealing with them. The application of this score may have a good impact on the safety of medical personnel.

In COVID-19, different grades of multiple B-lines with patchy distribution could be seen. Distance between B-lines is so variable that it could be distanced or confluent until the appearance of ‘white lung’ [14]. Some case reports described the findings of LUS in confirmed COVID-19 patients and reported an irregular pleural line with small subpleural consolidations, areas of white lung, and thick, confluent and irregular vertical artifacts (B-lines) [13]. Although our results carry variable findings, however, our results found that areas of consolidation in LUS are independent predictors for COVID-19 infection. LUS was reported to have 86% diagnostic accuracy in detecting alveolar consolidation and was able to differentiate between effusion and consolidation. Its specificity for detecting consolidation reached 100% in some studies [10].

Regarding the prognosis of COVID-19 infected patients, we analyzed the predictors for mortality. Old age, presence of cardiac problems and hypoxia on admission are clinical predictors of mortality. This is concordant with previous reports [6, 16].

Concerning LUS, we presented imaging predictors for mortality using this simple, safe, and cheap tool in COVID-19 patients. In our study, B-LUS profile and areas of consolidation were associated with mortality. Areas for consolidation was also correlated with prolonged hospitalization. This may also direct the medical staff to determine patients needing high care and those needing a long hospital stay.

Regarding other investigations, CT chest shows more consolidation and ground glass appearance in COVID-19 infected patients. This is consistent with other reports showing prominent radiologic abnormalities were bilateral ground-glass opacity and subsegmental consolidation areas [7, 17].

Our laboratory findings showed that COVID-19 infected patients had less blood O_2_ saturation and less lymphocytic count than other causes of pneumonia. However, in our study, lymphopenia has not reached a significant degree of correlation with mortality. Other reports showed the predominance of lymphopenia in COVID-19 infection among critical than other less critical patients and was associated with a severe course [6, 18]. A study conducted by Zhou et al. [6], found that baseline lymphocyte count was significantly higher in survivors than non-survivors of COVID-19 infected patients. However, in survivors, lymphocyte count improved after the 1st week of illness, whereas severe lymphopenia remained until death in non-survivors.

This study had some limitations. First, small number of patients is the main limitation of our study. Second, our score validity was tested on the same study cases, so we recommend further large studies in other centers in different situations to validate our ultrasound score.

## Conclusions

In conclusion, the application of our new score can stratify patients presented to ED with suspected COVID-19 pneumonia, considering that it is a good negative test. Moreover, this score may have a good impact on the safety of medical personnel.

## Data Availability

all data and materials are available.

## Abbreviations

COVID-19: Coronavirus disease 2019
SARS-Cov-2: Severe acute respiratory syndrome coronavirus 2
LUS: Lung ultrasound
NCIP: Novel coronavirus infected pneumonia
CFR: Case fatality rate
GGO: Ground-glass opacity
RT-PCR: Real-time reverse transcriptase-polymerase chain reaction
